# Changes in Flow Density Measured Using Optical Coherence Tomography Angiography after iStent Insertion in Combination with Phacoemulsification in Patients with Open-Angle Glaucoma

**DOI:** 10.1155/2018/2890357

**Published:** 2018-01-31

**Authors:** Maged Alnawaiseh, Viktoria Müller, Larissa Lahme, Ralph Laurent Merté, Nicole Eter

**Affiliations:** Department of Ophthalmology, University of Muenster Medical Center, Muenster, Germany

## Abstract

**Purpose:**

To evaluate changes in flow density after the implantation of a trabecular microbypass stent (iStent) in combination with cataract surgery.

**Methods:**

A total of 48 eyes of 48 patients, who underwent either cataract surgery alone (cataract group) or cataract surgery with implantation of two iStent inject devices (iStent group), were prospectively included in this study. Intraocular pressure (IOP) and flow density data before and after surgery were extracted and analyzed.

**Results:**

In the iStent group, the mean IOP was 18.2 ± 3.3 mmHg prior to surgery and 13.2 ± 2.3 at follow-up, and this difference was statistically significant (*p* < 0.001). The mean IOP in the cataract group also improved significantly after surgery (before: 17.1 ± 2.4; after: 15.1 ± 2.7 *p* = 0.003). The flow density (whole en face) in the superficial and deep retinal OCT angiogram of the macula (superficial: *p* = 0.002; deep: *p* = 0.034) and in the ONH (*p* = 0.011) improved significantly after surgery in the iStent group. The differences in the cataract group were not significant.

**Conclusions:**

Flow density of the macula and ONH, as measured by OCTA, improved significantly after cataract surgery with iStent. Noninvasive quantitative analyses of flow density provide a new parameter, which can help for the monitoring of therapy success after glaucoma surgery.

## 1. Introduction

Glaucoma is a leading cause of irreversible blindness worldwide, and its prevalence is projected to rise in the future. The treatment of glaucoma is based on a lowering of intraocular pressure to minimize the risk of visual loss [1, 2].

Microinvasive glaucoma surgery (MIGS) has attracted increasing interest in recent years. The microbypass stent or iStent is a small intraocular implant, which is inserted ab interno, sits within Schlemm canal, and reduces the IOP in mild to moderate glaucoma combined with a favorable safety profile [3–6]. iStent implantation is often performed concurrently with phacoemulsification, and the combined operation has been shown to significantly outperform phacoemulsification alone in the lowering of IOP [4] and to be similar to cataract surgery in terms of associated complications [5].

Optical coherence tomography angiography (OCTA) is a new imaging technique, which enables visualization of blood flow in the retina and optic nerve head without intravenously injected dye and has been described in healthy subjects in various retinal diseases and in animal models [7–12]. It is also possible to quantify the blood flow in the retina and ONH using OCTA, and a number of studies have demonstrated a reduced disc perfusion in patients suffering from glaucoma with this imaging procedure [9, 13, 14].

The aim of this study is to evaluate the impact of iStent insertion in combination with phacoemulsification on the flow density of the macula and ONH as measured using OCTA.

## 2. Materials and Methods

### 2.1. Subjects and Selection Criteria

Twenty-four consecutive patients diagnosed with cataract and open-angle glaucoma whose IOP was uncontrolled using their antiglaucoma medication were prospectively included in this study. The study followed the tenets of the Declaration of Helsinki and was approved by the Ethics Committee of the University of Muenster, North Rhine Westphalia, Germany. A control group of 24 eyes of 24 patients who underwent phacoemulsification cataract surgery without iStent implantation was also included.

Patients with glaucoma other than open-angle glaucoma, peripheral anterior synechiae, media opacities preventing a gonioscopic view of the angle or high-quality imaging, dense cataract, vitreoretinal disease, or neurological disease were excluded from the study.

Surgery was performed under topical or general anesthesia. In the cataract group, patients underwent a standard clear corneal phacoemulsification with implantation of a foldable IOL. In the iStent group, the standard clear corneal phacoemulsification was followed by an iStent implantation (Glaukos Corporation, Laguna Hills, CA). The surgical technique has been described in previous publications. In brief, after performing a standard clear corneal phacoemulsification with implantation of a foldable IOL, acetylcholine was injected into the anterior chamber to constrict the pupil. Next, the anterior chamber was filled with a viscoelastic agent (Healon, Abbott Medical Optics, Santa Ana, California, USA) to improve visualization of the angle and then, under gonioscopic view, two iStents were implanted through the trabecular meshwork into Schlemm's canal [5, 15].

### 2.2. Examination

All patients underwent a complete ocular examination including refraction, IOP measurement (Goldmann applanation tonometer), slit lamp biomicroscopy, gonioscopy, funduscopy, and OCT angiography imaging before and after surgery. OCT angiography imaging was obtained using the AngioVue OCTA system (RTVue XR Avanti with AngioVue, Optovue Inc, Fremont, California, USA). The system has an A-scan rate of 70,000 scans per second using a light source centered on 840 nm and a bandwidth of 45 nm. The split-spectrum amplitude-decorrelation angiography (SSADA) algorithm was used to extract the OCT angiography information. OCTA visualizes blood flow by means of technology described in detail in various studies in the literature. To visualize flow, OCT scans of a certain region are performed repeatedly and resultant OCT images are evaluated for changes. Whereas the blood flow in the retinal vessels will result in changes between subsequent OCT images, static tissue will show no change [8, 10, 16].

All OCTA imaging was performed under the same setting at the same location by an expert examiner and before imaging patients were asked to take a rest of about 5 minutes [17]. Macula imaging was performed using 3.0 × 3.0 mm scans while images over the optic nerve head were performed with 4.5 × 4.5 mm scans. The software automatically segmented the tissue into 4 layers, in the macula (superficial, deep, outer retina, and choriocapillaris) and in the ONH (optic nerve head, vitreous, radial peripapillary capillary (RPC), and choroid). The segmentations of all examinations were checked, and the flow density data of the optic nerve head and the macula were then extracted and analyzed. The flow density data were evaluated in the superficial retinal OCT angiogram of the macula, the deep retinal OCT angiogram of the macula, and the radial peripapillary capillary (RPC) layer of the optic nerve head (ONH). Images with lines or gaps arising from poor signal strength or motion artifacts were not included in the study.

### 2.3. Data Analysis and Statistics

Data management was performed using Microsoft Excel 2013. IBM SPSS® Statistics 22 for Windows (IBM Corporation, Somers, NY, USA) was used for statistical analyses. The normality of the data distribution was tested using the Kolmogorov-Smirnov test. After confirmation of the normality assumption, data are generally presented as mean ± standard deviation and changes at subsequent follow-up compared with baseline were assessed using paired sample *t*-tests. The two treatment groups were compared using independent Student's *t*-tests. The global statistical significance level was set to 0.05. All inferential statistics are intended to be exploratory, not confirmatory, and are interpreted accordingly.

## 3. Results

In this prospective study, 24 eyes of 24 patients with cataract and open-angle glaucoma (age 73.5 ± 6.2 years, 14 female, 10 male) were consecutively enrolled in the iStent group. Another 24 eyes of 24 patients (age 72.8 ± 8.9 years, 14 female, 10 male) were enrolled in the cataract group. There was no significant difference in age (*p* = 0.770) between the two groups.

In the iStent group, the mean IOP was 18.2 ± 3.3 mmHg prior to surgery and 13.2 ± 2.3 at follow-up, and this difference was statistically significant (*p* < 0.001). The IOP in the cataract group also improved significantly after surgery (before: 17.1 ± 2.4; after: 15.1 ± 2.7; *p* = 0.003). There was no statistically significant difference in either groups between the preoperative and postoperative signal strength index (SSI) (macula: iStent group: before: 62.8 ± 7.3; after: 63.4 ± 7.9; *p* = 0.70; cataract group: before: 59.4 ± 6.0; after: 61.4 ± 7.0; *p* = 0.14; ONH: iStent group: before: 57.7 ± 8.0; after: 58.3 ± 9.5; *p* = 0.87; cataract group: before: 56.3 ± 8.0; after: 60.9 ± 8.3; *p* = 0.06).

In the iStent group, the flow density (whole en face), as measured in the superficial and deep OCT angiogram of the macula and in the RPC of the ONH, improved significantly after surgery (superficial OCT angiogram: before: 44.6 ± 2.9; after: 47.6 ± 4.5; *p* = 0.002; deep OCT angiogram: before: 50.9 ± 3.6; after: 53.0 ± 4.2; *p* = 0.034; RPC: 43.5 ± 7.7; after: 45.4 ± 6.5). The flow density data of the iStent group are summarized in Table 1.

In the cataract group, there was no statistically significant difference between the preoperative and postoperative flow density whole en face (before surgery: 46.2 ± 2.5; after surgery: 47.1 ± 2.6; *p* = 0.20). The flow density data in the cataract group before and after surgery are summarized in Table 2.

## 4. Discussion

The flow densities in the retinal OCT angiogram of the macula and in the radial peripapillary capillary (RPC) network, as measured using OCTA, improved significantly after iStent implantation in conjunction with cataract surgery.

The iStent has become an important player among microinvasive glaucoma surgeries. Compared with filtrating surgery, the procedure has a higher safety profile and shorter recovery time and is sparing of conjunctival tissue, should a more invasive procedure be necessary [5, 18]. Microinvasive glaucoma surgery (MIGS) is therefore becoming more popular. However, the reduction in intraocular pressure seen with the iStent is lower than that achievable with filtering surgery [5]. In clinical practice, this minimally invasive procedure is usually performed in combination with cataract extraction. This study demonstrates the “typical” clinical use of the device and explores the utility of OCTA in monitoring the success of a given treatment.

Optical coherence tomography (OCT) angiography is a new imaging technique, in which the retinal and choroidal circulation can be visualized without contrast agent. OCTA also enables a quantitative analysis of blood flow. The reproducibility and repeatability of flow density data as measured by OCT angiography have been evaluated in normal subjects and in glaucoma patients in different studies in the literature [13, 19, 20].

Evaluation studies on OCTA in patients suffering from glaucoma show a reduction of ONH and macula perfusion in glaucoma patients compared with healthy controls. The flow density data also correlate with disease severity as well as functional and structural damage [9, 21, 22]. Furthermore, Akil et al. demonstrated that vessel density measurements derived from noninvasive OCT angiography show a stepwise decrease from normal eyes to preperimetric glaucoma eyes to mild POAG eyes [22]. Holló demonstrated that OCT angiography is also able to detect transient changes in peripapillary perfusion noninvasively in glaucoma patients. In that study, the peripapillary flow density was evaluated in 6 eyes of 4 patients with IOP > =35 mmHg before and after topical treatment. In Holló's case series, the peripapillary flow density increased significantly after medical IOP reduction in all cases [23]. Our study evaluated the impact of iStent combined with phacoemulsification in coexistent open-angle glaucoma and cataract on flow density measured using OCTA. To the best of our knowledge, this is the first study to evaluate the impact of a surgical lowering of IOP on flow density measurements. After cataract surgery in combination with iStent insertion, the vessel density (whole en face) improved significantly in the superficial retinal OCT angiogram of the macula, in the deep retinal OCT angiogram of the macula, and in the radial peripapillary capillary (RPC) layer of the optic nerve head (ONH). Although the IOP improved significantly after surgery in the cataract group, the differences of flow density were not significant. This may be explained by the relatively minor changes in IOP in the cataract group compared with the iStent group or by the small sample size.

This study is not without limitations. First, the image quality could be improved after cataract surgery, which might influence the flow density measurements. However, patients with media opacities preventing high-quality imaging and those with dense cataract were excluded from the study. In this context, it is also important to mention that there was no statistically significant difference between the pre- and postoperative signal strength index. Second, our study is also limited by its small sample size, and this should be considered when evaluating the outcome in the cataract group. However, this study was designed to evaluate the impact of iStent insertion in combination with phacoemulsification on flow density measurements and not to compare cataract surgery alone with cataract surgery in combination with iStent. This has been evaluated in other studies in the literature. Third, our results may have been affected by the short follow-up time. Further longitudinal studies involving larger numbers of patients are thus needed.

In conclusion, iStent insertion in combination with cataract extraction induced a significant improvement in macular and ONH perfusion. Not only does flow density, as measured by OCTA, appear to correlate with structural and functional glaucoma damage, but OCTA is also able to visualize acute changes in macula and ONH perfusion and can therefore be used to evaluate short-term therapy success.

## Figures and Tables

**(a):** 

iStent group	
*N*	24
Age (years)	73.46 ± 6.24
Gender (female : male)	14 : 10

**(b):** 

	Before surgery mean ± SD	After surgery mean ± SD	*p* value
IOP	18.17 ± 3.25	13.21 ± 2.34	**<0.001**
*OCTA—superficial*			
Flow density (whole en face)	44.56 ± 2.89	47.61 ± 4.46	**0.002**
Flow density (fovea)	29.50 ± 5.42	31.99 ± 7.06	**0.016**
Flow density (parafovea)	46.62 ± 3.11	49.52 ± 4.53	**0.003**
*OCTA—deep*			
Flow density (whole en face)	50.86 ± 3.62	52.97 ± 4.22	**0.034**
Flow density (fovea)	29.21 ± 6.22	31.47 ± 8.24	0.063
Flow density (parafovea)	53.05 ± 4.37	54.89 ± 4.63	0.074
*OCTA—RPC*			
Flow density (whole en face)	43.53 ± 7.70	45.40 ± 6.54	**0.011**
Flow density (inside disc)	25.83 ± 12.50	28.58 ± 14.36	**0.012**
Flow density (peripapillary)	52.49 ± 8.32	53.17 ± 6.89	0.421

**(a):** 

Cataract group	
*N*	24
Age (years)	72.79 ± 8.88
Gender (female : male)	14 : 10

**(b):** 

	Before surgery mean ± SD	After surgery mean ± SD	*p* value
IOP	17.14 ± 2.36	15.10 ± 2.74	**0.003**
*OCTA—superficial*			
Flow density (whole en face)	46.22 ± 2.49	47.05 ± 2.59	0.200
Flow density (fovea)	28.18 ± 5.45	28.57 ± 4.87	0.740
Flow density (parafovea)	48.63 ± 2.56	49.07 ± 3.05	0.576
*OCTA—deep*			
Flow density (whole en face)	53.08 ± 2.42	54.20 ± 2.28	0.099
Flow density (fovea)	29.45 ± 6.78	31.15 ± 6.64	0.333
Flow density (parafovea)	55.99 ± 2.63	56.30 ± 3.62	0.668
*OCTA—RPC*			
Flow density (whole en face)	50.16 ± 4.81	50.04 ± 4.58	0.827
Flow density (inside disc)	39.95 ± 9.89	40.77 ± 9.39	0.294
Flow density (peripapillary)	58.73 ± 6.16	57.90 ± 5.73	0.228
